# The Radcliffe Infirmary, Oxford

**Published:** 1891-05-02

**Authors:** 


### THE RADCLIFFE INFIRMARY, OXFORD.

In the last issue of the *Oxford Chronicle* the following article appears on the Radcliffe Infirmary: The controversy about the condition of the Radcliffe Infirmary is being conducted with a good deal of spirit on both sides. The promptitude with which the medical staff issued their reply to the report of The Hospital's Commissioner is equalled by the readiness of that gentleman in publishing his vindication. He declares in last week's Hospital that "our main criticisms are left practically untouched." We are, of course, as we have already said, unwilling to discuss any matters of a technical nature, but it does not require any special knowledge to see the force of some of the points which The Hospital's investigator makes against the Radcliffe Infirmary. "The general aspect which it presents to the visitor on entering" is obvious to the hundreds of people who daily pass along the Woodstock Road. Nor can it be said that any reply is forthcoming to the charge of insufficiency in the accommodation for the nurses. As to the interest in the Infirmary shown by the public, let us hear what the Committee of Management have to say in their report for the year 1890: "With regard to the receipts your Committee have to regret a falling off in the subscriptions to the amount of nearly £200. The deficiency was indeed chiefly made up by the greater liberality shown by the public, especially in the country districts, in their Hospital Sunday contributions. But even so the favourable balance with which last year closed has disappeared, and there remains in the ordinary receipt and expenditure account a deficit of a few pounds." So that, if we take a falling off in subscriptions to indicate a falling off in the sympathy, there really is some truth in the accusation. The probable fact of the matter is that the Infirmary building is rapidly becoming antiquated and obsolete of type. At any rate, it seems to require extensive and expensive alterations, and it is admitted that a good deal more money could advantageously be spent. Yet, surely, even if a new hospital should be thought desirable for Oxford, there ought to be no difficulty about ways and means. Oxford is a fairly wealthy city, and members of the University would also, no doubt, contribute liberally. No one in Oxford seriously doubts that good use is being made of the present building; but if a new one is really wanted, by all means let us have it, and not go on patching up from time to time an inefficient survival from another age. [A report of the Special Meeting of Governors called to consider our Commissioner's report will be found on page 55.]

